# Affective and Cognitive Distortions-Aided Suicide Risk Prediction for Long-Form Speech in Psychological Support Hotlines

**DOI:** 10.3390/bioengineering13060673

**Published:** 2026-06-10

**Authors:** Changwei Song, Jianqiang Li, Qing Zhao, Yining Chen, Yongsheng Tong, Guanghui Fu

**Affiliations:** 1 School of Computer Science, Beijing University of Technology, Beijing 100124, China; songchangwei@emails.bjut.edu.cn (C.S.); lijianqiang@bjut.edu.cn (J.L.) chenyn0317@emails.bjut.edu.cn (Y.C.); 2 Beijing Suicide Research and Prevention Center, Beijing Huilongguan Hospital, Beijing 100096, China; timystong@pku.org.cn; 3 Hôpital de la Pitié Salpêtrière, AP-HP, Institut du Cerveau (ICM, Paris Brain Institute), CNRS, Inria, Inserm, Sorbonne Université, 75013 Paris, France; guanghui.fu@inria.fr

**Keywords:** suicide risk prediction, long-form speech, psychological support hotline, graph attention network

## Abstract

Speech-based suicide risk prediction is vital for psychological support hotlines but remains challenging because existing methods often insufficiently incorporate clinically relevant prior cues and have difficulty identifying sparse high-risk signals in long-form speech. We propose the Affective & Cognitive Distortions-assisted Speech Suicide Risk Prediction Network (ACD-SSRNet) to address these challenges. First, we construct a multi-view feature system that integrates general acoustic-textual features with affective and cognitive-distortion cues motivated by clinical knowledge. Second, a hierarchical cascaded decoupling module is developed to reduce heterogeneous feature redundancy while preserving task-critical information. Finally, we design a prior-guided multi-path graph attention structure to locate sparse high-risk segments and capture long-range temporal dependencies. Experiments on a real-world hotline dataset show that ACD-SSRNet outperforms state-of-the-art baselines, achieving a 2.79% improvement in F1-score and a 2.57% improvement in accuracy. We further conducted an expert evaluation on five representative de-identified hotline cases, showing that the model can capture key affective and cognitive-distortion segments associated with suicide risk.

## 1. Introduction

Globally, approximately 703,000 people die by suicide each year, and suicide has become the second leading cause of premature death among young people aged 15–29 years [1], highlighting the urgent need to build an effective suicide prevention system. As a core component of suicide prevention and control, psychological support hotlines have been integrated into national mental health service networks in many countries [2,3]. By bridging individuals in crisis with professional counselors, hotlines provide immediate telephone support, systematic risk assessment, and timely intervention, serving as a critical frontline for suicide crisis intervention [4]. However, traditional suicide risk assessment based on clinical scales has obvious limitations in hotline scenarios: the reliability and validity of assessment results are highly dependent on the professional experience and operational proficiency of counselors, making it difficult to achieve standardized, real-time, and objective risk screening for massive hotline calls. To address this gap, speech-based automatic suicide risk prediction technology, which can mine risk-related abnormal patterns from natural conversation speech, is poised to become a research hotspot in the field of psychological crisis intervention.

Recent studies have advanced speech-based suicide risk prediction and shown its potential for hotline screening. However, several limitations still restrict its clinical usefulness. First, existing feature frameworks lack a clinically customized multi-view system anchored to core pathological markers of suicide risk: most studies rely heavily on domain-general statistical acoustic features (e.g., MFCC [5], COMPARE [6], eGeMAPS [7]) or deep representations from off-the-shelf speech foundation models (e.g., Wav2vec2 [8], Whisper [9]), which are not optimized for suicide risk assessment and have insufficient task adaptability and discriminative power decoupled from clinical diagnostic logic; although some studies have incorporated affective features as core predictive dimensions [10], nearly all overlook cognitive-distortion-related cues that are clinically relevant to suicide risk assessment, resulting in the complete absence of clinical prior anchors. Second, existing multi-feature fusion frameworks lack task-adapted solutions to eliminate heterogeneous feature redundancy: most methods [10,11,12] directly integrate multi-view features via simple concatenation or attention mechanism fusion, without feature decoupling preprocessing to eliminate inter-modal redundancy and noise, which directly masks faint and sparse high-risk discriminative signals; while shared-private architecture-based decoupling methods [13,14,15] can effectively solve multi-feature redundant entanglement, they adopt a peer-to-peer paradigm treating all features as equal independent views, with the number of constraint functions growing squarely with feature categories, which causes severe constraint combinatorial explosion when extended to 4+ feature types. Third, existing modeling paradigms are misaligned with standardized clinical suicide risk assessment principles, leading to poor capture of sparse high-risk segments in long-form speech: real-world psychological hotline calls typically last 20–120 min (typical long-duration speech), yet most methods are developed and validated on short clips (≤2 min), and even with session-level prediction via voting mechanisms [11,16,17], they overlook critical long-range temporal contextual information; while prior work like Song et al. [18] advanced long-form speech modeling via 30 s segment splitting and Transformer-LSTM temporal fusion, their framework lacks clinical pathological prior guidance, and as high-risk marker-carrying segments are often sparse and diluted by large volumes of non-high-risk content, existing general temporal modeling methods cannot perform targeted, targeted continuous modeling guided by clinically motivated priors and adaptive weighting, resulting in insufficient extraction of key high-risk discriminative information and loss of clinically meaningful long-range temporal logic.

To address the aforementioned three core limitations, we propose a novel Affective & Cognitive Distortions-assisted Speech Suicide Risk Prediction Network (ACD-SSRNet), with three targeted designs that directly correspond to the identified research gaps: First, we build a clinically informed multi-view feature system. It combines general acoustic-textual representations with affective and cognitive-distortion cues as prior anchors. Second, we design a hierarchical cascade decoupling module, which avoids the constraint of the combinatorial explosion defect of mainstream peer-to-peer decoupling paradigms when handling 4 feature categories, effectively eliminates inter-feature redundant entanglement while preserving task-critical complementary information, and adapts to the small-sample, high-stakes characteristics of suicide risk prediction. Third, we propose a pathological prior-guided multi-path graph attention learning framework, which better aligns model learning with clinically motivated risk cues, enables accurate localization of sparse high-risk segments in long-form hotline speech, and retains clinically meaningful long-range temporal dependencies. Experiments on a real-world psychological hotline dataset show that our ACD-SSRNet outperforms state-of-the-art baselines, with relative improvements of 2.79% in F1-score and 2.57% in accuracy. In addition, we conducted an expert evaluation and analyzed five representative cases using attention-based interpretability analysis, indicating that the model focuses on clinically plausible affective and cognitive cues.

The main contributions of this study are summarized as follows:

- Clinically motivated feature system innovation: We built a clinically informed multi-view feature system anchored to suicide-risk-related affective and cognitive cues, integrating affective and cognitive distortion features as prior anchors to improve task-specific discriminative power.
- Feature decoupling paradigm innovation: We proposed a hierarchical cascaded decoupling module that avoids direct all-to-all pairwise decoupling across all feature types and provides a more scalable structured alternative to peer-to-peer shared-private decoupling when multiple clinical prior features are introduced.
- Clinically aligned modeling innovation: We designed a pathological prior-guided multi-path graph attention learning framework, which constructs graph topology based on emotion labels and cognitive distortion labels, addresses the core limitation of existing methods that fail to capture sparse high-risk segments in long-form speech, and significantly improves the model’s prediction performance in real-world psychological hotline scenarios.

## 2. Related Work

### 2.1. Speech Suicide Prediction Based on Artificial Intelligence

In recent years, speech-based automatic suicide risk prediction has attracted extensive research attention for its non-invasiveness, accessibility, and low cost, demonstrating great potential for real-time screening in psychological support hotline scenarios. Existing studies have mainly explored this field along two core dimensions: feature extraction framework design and predictive modeling methodology. However, two critical unresolved limitations severely hinder their clinical translation, which are the core gaps addressed in this work: (1) the lack of a clinically customized multi-view feature system anchored to suicide risk pathological markers; (2) the absence of a task-adapted multi-feature fusion and decoupling paradigm for suicide risk prediction.

For feature extraction, existing studies have widely explored multi-source feature fusion to enhance representation capability for suicide risk assessment. Amiriparian et al. [11] first proposed a multi-feature fusion strategy integrating statistical acoustic features (eGeMAPS) [7], deep spectral features (DEEPSPECTRUM) [19], and wav2vec2 [8] pre-trained embeddings, verifying the complementary value of multi-modal features for this task. Subsequent works further optimized feature extraction with large-scale pre-trained speech models: Cui et al. [20] and Song et al. [18] adopted Whisper [9] for deep speech feature extraction with excellent performance in content and prosody modeling; Chen et al. [10] further introduced Emotion2Vec [21] affective embeddings to improve the pertinence of affective information, marking an important advance in task-specific feature optimization. Despite these advances, existing feature frameworks have a fundamental unresolved limitation (the first core gap of this work): nearly all are built on domain-general speech representations without task-specific customization aligned with the clinical diagnostic logic of suicide risk. Most critically, existing studies universally overlook cognitive distortion features—the clinically validated core pathological markers of suicide risk. Even works incorporating affective features fail to establish a multi-view feature system anchored to clinical pathological priors, resulting in insufficient discriminative power for high-risk sample identification.

For predictive modeling, existing studies have gradually established a standard paradigm of “speech segmentation—feature extraction—predictive modeling—result aggregation”. Early works represented by Scherer et al. [16] and Belouali et al. [17] pioneered this pipeline by segmenting long speech into fixed-duration clips, performing clip-level prediction with traditional machine learning, and aggregating results via voting. Amiriparian et al. [11] further optimized segmentation with WhisperX-based [22] sentence-level cutting to avoid semantic fragmentation but still used SVM for clip-level prediction with simple score averaging, failing to capture inter-segment temporal dependencies. To address temporal modeling limitations, deep learning methods have been widely introduced in recent years. Ding et al. [12] constructed a prediction model based on Bi-LSTM and self-attention to capture temporal dependencies, while Song et al. [18] further advanced long-form speech modeling by splitting speech into 30 s clips, extracting features via Whisper, and implementing temporal fusion with LSTM and Transformer. However, existing methods have a second core unresolved limitation: the lack of an effective multi-feature fusion and decoupling design adapted to suicide risk prediction. Most existing fusion pipelines only use simple concatenation, attention-weighted fusion, or result-level voting, without decoupling preprocessing to eliminate heterogeneous feature redundancy. For suicide risk prediction, high-risk discriminative signals are often faint and sparse in speech, and unprocessed feature redundancy will directly overwhelm these weak signals, severely degrading high-risk recognition performance. Meanwhile, mainstream decoupling paradigms (e.g., shared-private architectures) are inherently unsuitable for this task: they adopt a peer-to-peer paradigm where the number of constraint functions grows squarely with the number of feature categories, triggering severe combinatorial explosion when handling 4+ feature types (consistent with our clinically customized multi-view system).

### 2.2. Graph Topological Structure of Long Speech Sequences

In recent years, graph neural networks (GNNs) have been widely adopted in analytical frameworks for long-duration speech classification tasks. These methods typically segment long speech into sentence-level units, treat each unit as a node in a graph, and model inter-node relationships to capture contextual information. However, existing GNN-based approaches still have a critical limitation: irrationality in graph structure construction, which prevents effective capture of the intrinsic characteristics of long-duration speech required for suicide risk prediction. Specifically, Chen et al. [23] constructed a graph based solely on temporal relationships between speech segments, while Sheikh et al. [24] built their graph using feature similarity and the Top-K mechanism—both relying on a single correlation criterion for graph construction. Even models incorporating emotional information still adopt simplistic graph construction strategies: Zhou et al. [25] filtered graph edges only based on emotional label consistency, while Yu et al. [26], despite focusing on emotional features, did not address the core flaw of single-criterion graph construction. The core common limitation of these methods—corresponding to the third core research gap of this study—is the defects in graph structure construction. All existing approaches rely solely on single correlation criteria (e.g., temporal relationships, feature similarity, emotional label consistency) and fail to comprehensively consider the abundant contextual and temporal relationships in long speech sequences. This flaw severely limits the graph model’s ability to capture the intrinsic structure of speech, making it unable to identify sparse high-risk segments diluted in long-duration speech—an essential requirement for suicide risk prediction, which is the third core unresolved limitation targeted in this work.

## 3. Methods

**Figure 1:**
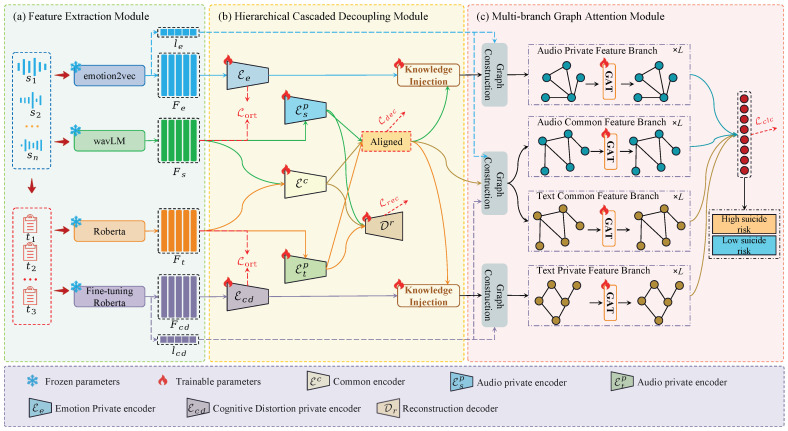
Overall architecture of the proposed Affective & Cognitive Distortions-assisted Speech Suicide Risk Prediction Network (ACD-SSRNet) for long-form speech suicide risk assessment in psychological support hotlines. The framework consists of three core modules: a feature extraction module, a hierarchical cascaded decoupling module, and a multi-branch graph attention module, which work collaboratively to capture and identify suicide risk signals in long-form speech.

This paper proposes an innovative framework for suicide risk prediction in long speech from psychological counseling hotlines, named Affective & Cognitive Distortions-assisted Speech Suicide Risk Prediction Network (ACD-SSRNet). As illustrated in Figure 1, the framework achieves accurate capture and recognition of suicide risk signals in long speech through the synergistic work of three core modules.

### 3.1. Problem Definition

We focus on the acoustic-text fusion-based suicide risk prediction task in psychological hotline scenarios. Given a dataset of long-form samples from real-world psychological support hotlines, denoted as the set $X=\{x_{1},x_{2},...,x_{N}\}$, where *N* is the total number of samples. Each sample $x_{i}$ corresponds to the complete data of a help-seeker, which consists of consecutive sentence-level speech units and their corresponding text units. Specifically, $x_{i}=\left\{\left(s_{i1},t_{i1}\right),\left(s_{i2},t_{i2}\right),\ldots ,\left(s_{in_{i}},t_{in_{i}}\right)\right\}$, where $s_{ij}$ represents the *j*-th sentence-level speech unit of the *i*-th sample, $t_{ij}$ denotes the text unit corresponding to $s_{ij}$, and $m_{i}$ is the total number of sentence-level units in the *i*-th sample. We set a pre-defined maximum length threshold of 256 for the input sequence of each sample, so the number of valid sentence-level units for each sample is capped at 256 (i.e., if $m_{i}>256$, only the first 256 units $\left(s_{ij},t_{ij}\right)$ are retained for modeling). For the brevity of subsequent descriptions, the sample index *i* can be omitted and denoted as $\left(s_{j},t_{j}\right)$ without confusion about sample attribution, where $s_{j}$ is the sentence-level speech unit and $t_{j}$ is its corresponding text unit. The core task of this study is to construct a deep learning model that fuses the speech features of $s_{ij}$ and text features of $t_{ij}$ within each complete sample $x_{i}$ to predict the corresponding suicide risk label $y_{i}\in \left\{0,1\right\}$ for each sample.

### 3.2. Feature Extraction Module

**Figure 2:**
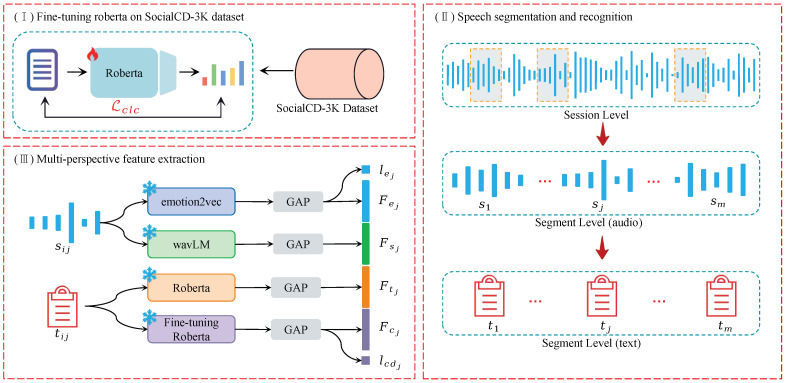
Detailed pipeline of the feature extraction module. It includes three key stages: fine-tuning the RoBERTa model on the SocialCD-3K dataset to obtain the cognitive distortion feature extractor (CD-RoBERTa), speaker diarization and speech-to-text conversion for hotline call recordings, and multi-perspective feature extraction to obtain general acoustic features, general text features, affective features, and cognitive distortion features simultaneously.

The feature extraction module is designed to capture multi-dimensional and complementary feature representations from speech and text modalities, laying a solid foundation for subsequent suicide risk prediction. As shown in Figure 2, feature extraction consists of three key steps.

Given the close association between cognitive distortions and suicide risk [27], we treat cognitive distortion as an important source of prior knowledge. We therefore fine-tune RoBERTa [28] on the SocialCD-3K [29] dataset to build CD-RoBERTa, which is used to identify distortion-related expressions and extract corresponding features. As the first Chinese multi-label classification dataset for cognitive distortion detection, SocialCD-3K comprises 3,407 Chinese social media posts, annotated with 12 types of cognitive distortions, including “All-or-nothing thinking” and “Over-generalization”.

For each long-form speech sample from psychological hotline scenarios, we first perform speaker segmentation using the speaker-diarization-community-1 (https://huggingface.co/pyannote/speaker-diarization-community-1 (accessed on 14 January 2026) [30,31,32] tool to separate the speech signals of the caller and the counselor. To focus on the emotional expression and psychological state of the caller, which are closely related to suicide risk assessment, only the caller’s speech segments are retained after segmentation. Subsequently, the retained caller’s speech segments are converted into corresponding text content using the SenseVoice (https://github.com/FunAudioLLM/SenseVoice (accessed on 14 January 2026)) tool. This speech-to-text conversion process ensures that we can obtain accurate text data corresponding to the caller’s speech, providing a basis for text-related feature extraction.

Based on the caller’s processed speech and text, we extract four distinct feature representations. Domain-general text features are obtained using the original RoBERTa model, where global average pooling (GAP) over the final Transformer layer output yields segment-level features $F_{t_{j}}\in R^{1\times d_{t}}$, and concatenation across all segments forms the sample-level feature $F_{t}\in R^{m\times d_{t}}$ with $d_{t}$ as the feature dimension. Cognitive distortion text features are extracted by CD-RoBERTa fine-tuned on SocialCD-3K, producing segment-level features $F_{cd_{j}}\in R^{1\times d_{t}}$ and sample-level features $F_{cd}\in R^{m\times d_{t}}$ along with cognitive distortion labels $l_{cd}$ through the same pooling operation. Domain-general speech features are extracted via WavLM [33], generating segment-level features $F_{s_{j}}\in R^{1\times d_{s}}$ from the final Transformer layer output and sample-level features $F_{s}\in R^{m\times d_{s}}$ after concatenation, where $d_{s}$ denotes the speech feature dimension. Emotion speech features are derived from Emotion2Vec, with global average pooling on the model output producing segment-level features $F_{e_{j}}\in R^{1\times d_{s}}$ and integrated sample-level features $F_{e}\in R^{m\times d_{s}}$ together with emotion labels $l_{e}$. In this study, the suicide-risk gold standard is the psychiatrist-confirmed 12-month follow-up outcome at the call level. By contrast, the cognitive distortion labels extracted by CD-RoBERTa are used as auxiliary clinically motivated prior cues for representation learning and graph construction, rather than as independent gold-standard supervisory labels for the hotline corpus.

### 3.3. Hierarchical Cascaded Decoupling Module

A key motivation for this hierarchical design is to avoid direct all-to-all pairwise decoupling across all feature types. Let *V* denote the total number of feature types. In a peer-to-peer decoupling paradigm, the number of direct pairwise cross-feature relations grows as V2=V(V−1)/2. In the current setting, V=4 (general speech, general text, affective prior, and cognitive-distortion prior), which would already correspond to six pairwise relations if all feature types were treated symmetrically. By contrast, the proposed HCDM decomposes the problem into two stages. The first stage only performs local prior-general decoupling for matched pairs, i.e., affective–speech and cognitive-distortion–text. The second stage then applies shared-private decoupling only to the two general modalities rather than to all four feature types jointly. More generally, if *r* additional clinical prior feature types are introduced on top of the two general modalities, a peer-to-peer formulation grows quadratically, whereas the proposed staged formulation only adds one local first-layer relation per new prior feature while keeping the second-layer structure unchanged. This is the sense in which the proposed design avoids constraint combinatorial explosion.

To address the redundant entanglement among multi-modal and multi-type features extracted in the previous module and enhance the discriminative power of features for suicide risk prediction, a Hierarchical Cascaded Decoupling Module is proposed. This module adopts a two-layer cascaded structure to sequentially realize the decoupling between domain-specific and domain-general features, as well as the decoupling between cross-modal general features, while integrating a knowledge injection mechanism to enhance feature representation.

**Figure 3:**
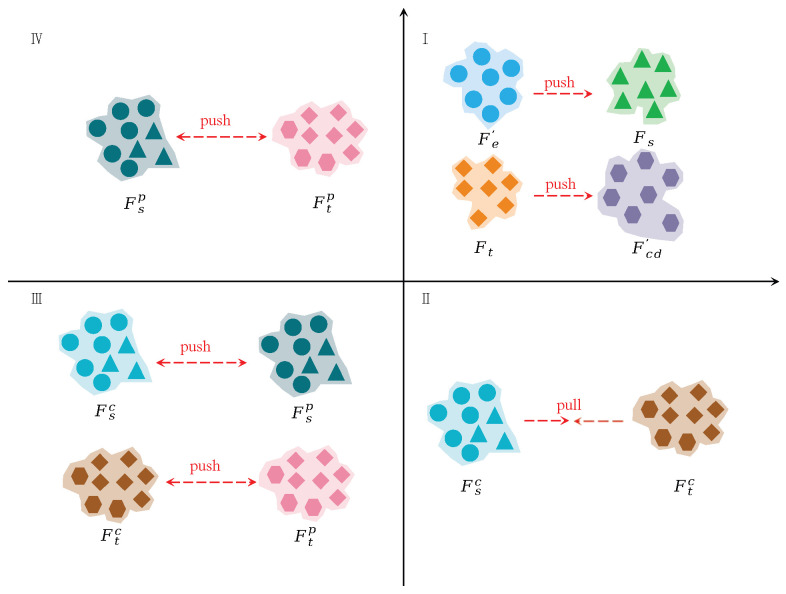
Illustration of the multi-component decoupling constraints in the Hierarchical Cascaded Decoupling Module. This figure summarizes four complementary constraint relationships rather than a complete forward computation flow. The (**I**) panel corresponds to the orthogonal decoupling between domain-specific prior features and their corresponding domain-general features; the (**II**) panel shows the shared feature consistency constraint between the speech and text shared representations; the (**IV**) panel enforces orthogonality between cross-modal private features; and the (**III**) panel enforces orthogonality between shared and private features within each modality. Together, these constraints reduce heterogeneous feature redundancy while preserving complementary suicide-risk-related information.

**Figure 4:**
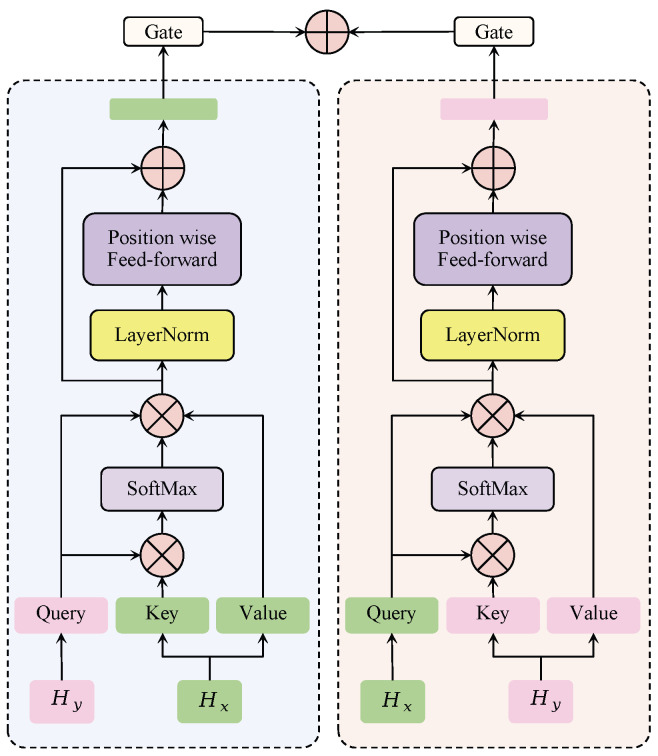
Structure of the knowledge injection mechanism. It adopts bidirectional cross-attention to fuse clinical prior knowledge (affective and cognitive distortion features) with general modality-specific features and uses adaptive gate units to dynamically balance the contribution of prior knowledge and general features, enhancing the clinical alignment and representation ability of the model.

For clarity, Figure 3 should be interpreted as a schematic summary of the constraint relationships used in the module, rather than as a left-to-right forward computation graph. The top-right panel belongs to the first decoupling layer and describes the separation between domain-specific prior features and their corresponding domain-general features. The remaining three panels correspond to the second-layer shared-private decoupling: the lower-right panel aligns the shared speech-text representations, the top-left panel separates the cross-modal private representations, and the lower-left panel separates the shared and private representations within each modality. The knowledge injection mechanism is presented separately in Figure 4.

#### 3.3.1. Decoupling of General and Domain-Specific Features

The first layer of the module focuses on decoupling domain-specific features from their corresponding domain-general features and enhancing general features through knowledge injection. Specifically, two lightweight fully connected layers are adopted as encoders to extract refined domain-specific feature representations: the emotion encoder $E_{e}$ for emotion features and the cognitive distortion encoder $E_{cd}$ for cognitive distortion features. The emotion feature $F_{e}$ and cognitive distortion feature $F_{cd}$ are encoded by $E_{e}$ and $E_{cd}$ to generate refined domain-specific features $F_{e}^{\prime }$ and $F_{cd}^{\prime }$, respectively, which are defined as

(1) $$F_{e}^{\prime }=E_{e}\left(F_{e};\theta_{e}\right)$$

(2) $$F_{cd}^{\prime }=E_{cd}\left(F_{cd};\theta_{cd}\right)$$

where $\theta_{e}$ and $\theta_{cd}$ denote the trainable parameters of $E_{e}$ and $E_{cd}$, respectively; $F_{e}^{\prime }\in R^{m\times d_{s}}$ is the refined emotion feature after encoding, and $F_{cd}^{\prime }\in R^{m\times d_{t}}$ is the refined cognitive distortion feature after encoding, with *m* representing the number of segment units, $d_{s}$ the speech feature dimension, and $d_{t}$ the text feature dimension.

To eliminate redundant entanglement between domain-specific and domain-general features, orthogonal loss is introduced for feature decoupling. As illustrated in the top-right section of Figure 3, the refined emotion feature $F_{e}^{\prime }$ is decoupled from the domain-general speech feature $F_{s}$, and the refined cognitive distortion feature $F_{cd}^{\prime }$ is decoupled from the domain-general text feature $F_{t}$. The orthogonal loss function minimizes the inner product between the two groups of features, thereby suppressing feature redundancy, which is defined as

(3) $$L_{ort}={∥F_{e}^{\prime }⊙F_{s}∥}_{F}^{2}+{∥F_{cd}^{\prime }⊙F_{t}∥}_{F}^{2}$$

where ${∥\cdot ∥}_{F}$ denotes the Frobenius and ⊙ denotes the Hadamard product.

#### 3.3.2. Cross-Modal Feature Decoupling

The second layer of the module focuses on decoupling the enhanced cross-modal general features $F_{t}$ and $F_{s}$, adopting a traditional feature decoupling method based on a shared-private encoder structure to separate shared and private information between text and speech modalities. All encoders in this layer are implemented as lightweight, fully connected layers to ensure efficiency and compatibility with feature decoupling objectives. Specifically, three encoders are designed: a shared encoder $E^{c}$ to extract cross-modal shared information, and two private encoders $E_{s}^{p}$ (for speech) and $E_{t}^{p}$ (for text) to extract modality-specific private information. The shared information reflects the common characteristics of text and speech related to suicide risk, while the private information retains the unique discriminative information of each modality. The encoding processes are defined as

(4) $$F_{s}^{c}=E^{c}\left(F_{s};\theta_{c}\right)$$

(5) $$F_{s}^{p}=E_{s}^{p}\left(F_{s};\theta_{s^{p}}\right)$$

(6) $$F_{t}^{c}=E^{c}\left(F_{t};\theta_{c}\right)$$

(7) $$F_{t}^{p}=E_{t}^{p}\left(F_{t};\theta_{t^{p}}\right)$$

where $\theta_{c}$, $\theta_{s^{p}}$, and $\theta_{t^{p}}$ denote the trainable parameters of $E^{c}$, $E_{s}^{p}$ and $E_{t}^{p}$, respectively; $F_{s}^{c}$ and $F_{t}^{c}$ are the shared features of speech and text modalities; $F_{s}^{p}$ and $F_{t}^{p}$ are the private features of speech and text modalities.

To ensure thorough and hierarchical feature decoupling, the total decoupling loss for the second layer is defined as the sum of three complementary constraint terms:

(8) $$L_{dec}=L_{consist}+L_{priv-ortho}+L_{intra-ortho}$$

where $L_{consist}$ denotes shared feature consistency loss, $L_{priv-ortho}$ denotes cross-modal private feature orthogonal loss, and $L_{intra-ortho}$ denotes intra-modal shared–private orthogonal loss. Together, these losses enforce effective separation between shared and private features as well as between cross-modal private features. More specifically, $L_{consist}$ constrains the shared speech and text representations to encode common suicide-risk-related information, $L_{priv-ortho}$ encourages the two private branches to retain complementary modality-specific cues, and $L_{intra-ortho}$ prevents the shared and private features within the same modality from collapsing into each other.

As depicted in the lower-right panel of Figure 3, the shared feature consistency loss is formulated to align cross-modal semantic representations in the latent feature space. Specifically, this loss enforces semantic consistency between the speech-derived shared features $F_{s}^{c}$ and text-derived shared features $F_{t}^{c}$ by maximizing their cosine similarity, which prioritizes the alignment of semantic orientation rather than absolute feature magnitude, thereby ensuring task-relevant information is consistently encoded across both modalities. This loss is formally defined as

(9) $$L_{\mathrm{consist}}=1-cos\left(F_{s}^{c},F_{t}^{c}\right)$$

where cos(·,·) denotes the cosine similarity operator between two feature vectors, formally defined as $\cos\left(a,b\right)=\frac{a^{⊤}b}{{∥a∥}_{2}{∥b∥}_{2}}$ for arbitrary feature vectors *a* and *b*, and ${∥\cdot ∥}_{2}$ represents the L2 norm.

As illustrated in the top-left corner of Figure 3, the private feature orthogonal loss eliminates redundant entanglement between modality-specific private features, encouraging $F_{s}^{p}$ and $F_{t}^{p}$ to capture unique and complementary information. It is defined as

(10) $$L_{priv-ortho}={∥F_{s}^{p}⊙F_{t}^{p}∥}_{F}^{2}$$

Furthermore, the intra-modal orthogonal loss is introduced to strengthen decoupling within each individual modality. As illustrated in the lower-left corner of Figure 3, it penalizes redundancy between shared and private features of the same modality, preserving their independence and distinctiveness. This loss is given by

(11) $$L_{intra-ortho}={∥F_{s}^{c}⊙F_{s}^{p}∥}_{F}^{2}+{∥F_{t}^{c}⊙F_{t}^{p}∥}_{F}^{2}$$

where ${∥\cdot ∥}_{F}$ denotes the Frobenius norm and ⊙ denotes the Hadamard product.

To further ensure the integrity of the encoded features and avoid the loss of task-relevant discriminative information, a reconstruction decoder $D_{r}$ is introduced in the second layer, along with a reconstruction loss. The reconstruction decoder implemented with fully connected layers $D_{r}$ takes the concatenated shared and private features of each modality as input and reconstructs the original general features $F_{s}$ and $F_{t}$, thereby constraining the encoder to learn feature representations that can fully retain the original information. The reconstruction process is defined as:

(12) $${\hat{F}}_{s}=D_{r}\left(\mathrm{concat}\left(F_{s}^{c},F_{s}^{p}\right);\theta_{dr}\right)$$

(13) $${\hat{F}}_{t}=D_{r}\left(\mathrm{concat}\left(F_{t}^{c},F_{t}^{p}\right);\theta_{dr}\right)$$

where $\theta_{dr}$ denotes the trainable parameters of the reconstruction decoder $D_{r}$; ${\hat{F}}_{s}$ and ${\hat{F}}_{t}$ are the reconstructed enhanced speech and text general features, respectively; concat(·) represents the feature concatenation operation. The reconstruction loss is formulated to guarantee that the reconstructed features faithfully recover the semantic information of the original enhanced features for both speech and text modalities. To this end, we adopt cosine similarity as the alignment metric, and this loss is computed as

(14) $$L_{\mathrm{rec}}=\left[1-\cos\left({\hat{F}}_{s},F_{s}\right)\right]+\left[1-\cos\left({\hat{F}}_{t},F_{t}\right)\right]$$

#### 3.3.3. Knowledge Injection

As shown in Figure 4, following intra-modal orthogonal decoupling, we employ bidirectional cross-attention to achieve mutual information fusion between the refined affective features $F_{e}^{\prime }$ and general speech features $F_{s}^{p}$, as well as between refined cognitive distortion features $F_{cd}^{\prime }$ and general text features $F_{t}^{p}$. Adaptive gate mechanisms are then applied to the two cross-attention outputs to balance the contributions of prior and general information. The knowledge injection process is formulated as

(15) $${\tilde{F}}_{s1}^{p}=\mathrm{CrossAtt}\left(F_{e}^{\prime },F_{s}^{p}\right),\;{\tilde{F}}_{s2}^{p}=\mathrm{CrossAtt}\left(F_{s}^{p},F_{e}^{\prime }\right)$$

(16) $${\tilde{F}}_{t1}^{p}=\mathrm{CrossAtt}\left(F_{cd}^{\prime },F_{t}^{p}\right),\;{\tilde{F}}_{t2}^{p}=\mathrm{CrossAtt}\left(F_{t}^{p},F_{cd}^{\prime }\right)$$

(17) $$g_{s}=\sigma \left(W_{s}F_{s}+b_{s}\right),\;g_{t}=\sigma \left(W_{t}F_{t}+b_{t}\right)$$

(18) $${F_{s}^{p}}^{\prime }=g_{s}⊙{\tilde{F}}_{s1}^{p}+\left(1-g_{s}\right)⊙{\tilde{F}}_{s2}^{p},\;{F_{t}^{p}}^{\prime }=g_{t}⊙{\tilde{F}}_{t1}^{p}+\left(1-g_{t}\right)⊙{\tilde{F}}_{t2}^{p}$$

where CrossAtt(·) denotes the cross-attention module, $g_{s},g_{t}$ are segment-level adaptive gate weights, and ⊙ denotes the Hadamard product.

Intuitively, the knowledge injection module is introduced after the first decoupling stage because the refined prior features and the modality-specific general features contain complementary information. The refined affective and cognitive-distortion features emphasize clinically relevant prior cues, whereas the modality-specific general features retain acoustic and semantic details that may not be fully represented by the priors alone. Bidirectional cross-attention enables each source to query the other from both directions so that clinically salient prior cues can highlight relevant modality-specific segments, while general features can preserve contextual details and reduce over-reliance on a single prior source. The adaptive gates further control the relative contribution of the two directions, preventing noisy or incomplete prior information from dominating the fused representation. In this way, feature interaction is guided and selective rather than simple concatenation.

### 3.4. Multi-Branch Graph Attention Module

To fully exploit the contextual dependencies and discriminative information among the decoupled features $F_{s}^{c}$, ${F_{s}^{p}}^{\prime }$, $F_{t}^{c}$, and ${F_{t}^{p}}^{\prime }$ from the Hierarchical Cascaded Decoupling Module, a Multi-branch Graph Attention Module is proposed. This module constructs distinct graph topological structures for each of the four features, respectively, to model the intrinsic associations between segment-level features, and then employs graph attention mechanisms to adaptively learn the importance of each node, ultimately fusing the features for suicide risk detection.

The core of this module lies in the construction of graph topological structures, where each feature corresponds to an independent graph branch, and the adjacency matrix of each graph is constructed based on the task-specific domain knowledge (emotion and cognitive distortion labels) extracted in the feature extraction stage. Specifically, the four graph branches are denoted as $G_{s^{p}}$, $G_{t^{p}}$, $G_{s^{c}}$, and $G_{t^{c}}$, corresponding to features ${F_{s}^{p}}^{\prime }$, ${F_{t}^{p}}^{\prime }$, $F_{s}^{c}$, and $F_{t}^{c}$, respectively. The node of each graph corresponds to the segment-level feature of the corresponding feature matrix (i.e., ${F_{s_{j}}^{p}}^{\prime }$, ${F_{t_{j}}^{p}}^{\prime }$, $F_{s_{j}}^{c}$, $F_{t_{j}}^{c}$ for the *j*-th node), and the edge construction rules of each graph are detailed as follows.

Clinically, suicide risk in hotline conversations is rarely expressed through a single isolated utterance; rather, it is reflected in the recurrence, persistence, and temporal evolution of affective disturbance and maladaptive cognition across different parts of the call. For this reason, we do not construct graph edges solely based on generic feature similarity. Instead, we use emotion labels and cognitive distortion labels as clinically motivated anchors: recurrent emotion patterns indicate sustained affective states, recurrent cognitive distortion labels indicate repeated maladaptive thinking patterns, and their co-occurrence captures clinically meaningful pathological coupling. Temporal adjacency is further introduced to preserve the local sequential continuity that is also important in real-world clinical assessment.

The rationale for constructing four graph branches instead of a single unified graph is that the four decoupled feature groups have different semantics after HCDM and therefore require different edge definitions. The speech-private branch focuses on affective continuity in acoustic expression, the text-private branch focuses on distortion-related textual risk cues, and the two shared branches capture clinically meaningful cross-modal common contexts. In the shared branches, we connect both low-risk supportive states (positive emotion without cognitive distortion) and high-risk pathological states (negative emotion with cognitive distortion), while temporal adjacency preserves local conversational evolution. This design allows the model to separately learn modality-specific and modality-shared relational patterns before late fusion, rather than forcing heterogeneous relations into a single graph with one uniform adjacency rule.

**Figure 5:**
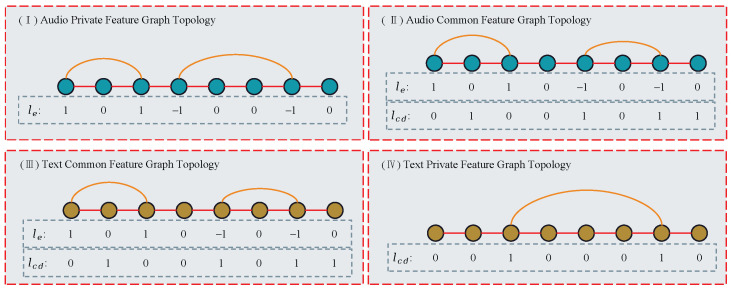
Four independent graph topological branches are constructed corresponding to speech private features, text private features, speech shared features, and text shared features, respectively. Each branch uses clinical prior labels (emotion and cognitive distortion) and temporal adjacency to build edges.

For the graph $G_{s^{p}}$ corresponding to the speech private feature ${F_{s}^{p}}^{\prime }$, the topological structure is constructed based on the emotion labels output by the Emotion2Vec model and the temporal adjacency of segments. Let $l_{e_{j}}\in \left\{-1,0,1\right\}$ denote the emotion label of the *j*-th segment, where $l_{e_{j}}=1$ represents positive emotion, $l_{e_{j}}=0$ represents neutral emotion, and $l_{e_{j}}=-1$ represents negative emotion. As illustrated in Part (I) of Figure 5, nodes are connected in two scenarios: one is nodes with the same emotion label, and the other is nodes that are adjacent in time sequence (i.e., consecutive segments), which ensures that the graph can capture both the contextual correlation of the same emotional tendency and the temporal dependency of speech private features. The adjacency matrix $A_{s^{p}}\in R^{m\times m}$ of $G_{s^{p}}$ is defined as

(19) $$A_{s^{p}}\left(i,j\right)=\left\{\begin{array}{cc} 1, & \mathrm{if}\;(l_{e_{i}}=l_{e_{j}}\;\mathrm{or}\;|i-j|=1)\;\mathrm{and}\;i\neq j,\\ 0, & \mathrm{otherwise}, \end{array}\right.$$

where $A_{s^{p}}\left(i,j\right)$ denotes the edge weight between the *i*-th and *j*-th nodes; *i* and *j* represent the index of segment-level nodes, and *m* is the total number of segment units. This rule is clinically motivated because affective disturbance in callers at suicide risk is often expressed intermittently rather than continuously. Two temporally separated segments with the same emotion label may therefore reflect the same underlying affective state. Connecting same-emotion segments enables the graph to aggregate distributed affective evidence, while temporal edges preserve short-range conversational continuity.

For the graph $G_{t^{p}}$ corresponding to the text private feature ${F_{t}^{p}}^{\prime }$, the topological structure is constructed based on the cognitive distortion labels output by the CD-RoBERTa model and the temporal adjacency of segments. Let $l_{{cd}_{j}}\in \left\{0,1\right\}$ denote the cognitive distortion label of the *j*-th segment, where $l_{c_{j}}=1$ indicates the presence of cognitive distortion and $l_{c_{j}}=0$ indicates the absence of cognitive distortion. As illustrated in Part (IV) of Figure 5, nodes are connected in two scenarios: one is nodes with cognitive distortion (i.e., $l_{{cd}_{j}}=1$), and the other is nodes that are adjacent in time sequence, to focus on both the contextual correlation of cognitive distortion-related information and the temporal dependency in text private features. The adjacency matrix $A_{t^{p}}\in R^{m\times m}$ of $G_{t^{p}}$ is defined as:

(20) $$A_{t^{p}}\left(i,j\right)=\left\{\begin{array}{cc} 1, & \mathrm{if}\;(l_{{cd}_{i}}=l_{{cd}_{j}}=1\;\mathrm{or}\;|i-j|=1)\;\mathrm{and}\;i\neq j,\\ 0, & \mathrm{otherwise}, \end{array}\right.$$

This rule is clinically motivated because cognitive distortions, such as over-generalization or all-or-nothing thinking, may recur across nonadjacent utterances within the same call. Linking distortion-positive segments allows the model to integrate dispersed evidence of maladaptive cognition that is highly relevant to suicide risk, while temporal edges preserve the local narrative progression of the caller’s thinking.

For the graphs $G_{s^{c}}$ and $G_{t^{c}}$ corresponding to the speech shared feature $F_{s}^{c}$ and text shared feature $F_{t}^{c}$, respectively, the topological structures are constructed based on the combination of emotion labels, cognitive distortion labels, and the temporal adjacency of segments, aiming to capture the common contextual correlation and temporal dependency of speech and text shared features related to suicide risk. As illustrated in Part (II) and Part (III) of Figure 5, nodes are connected in three scenarios: one is nodes with positive emotion and no cognitive distortion (i.e., $l_{e_{j}}=1$ and $l_{{cd}_{j}}=0$), the second is nodes with negative emotion and cognitive distortion (i.e., $l_{e_{j}}=-1$ and $l_{{cd}_{j}}=1$), and the third is nodes that are adjacent in time sequence. The adjacency matrices $A_{s^{c}}\in R^{m\times m}$ and $A_{t^{c}}\in R^{m\times m}$ of $G_{s^{c}}$ and $G_{t^{c}}$ are uniformly defined as:

(21) $$A_{s^{c}}\left(i,j\right)=A_{t^{c}}\left(i,j\right)=\left\{\begin{array}{cc} 1, & \mathrm{if}\;i\neq j,\;\mathrm{and}\;\mathrm{one}\;\mathrm{of}\;\mathrm{the}\;\mathrm{following}\;\mathrm{holds}\text{:}\\ \; & \begin{array}{cc} \; & \;\left(1\right)\;l_{e_{i}}=l_{e_{j}}=1\;\mathrm{and}\;l_{{cd}_{i}}=l_{{cd}_{j}}=0,\\ \; & \;\left(2\right)\;l_{e_{i}}=l_{e_{j}}=-1\;\mathrm{and}\;l_{{cd}_{i}}=l_{{cd}_{j}}=1,\\ \; & \;\left(3\right)\;|i-j|=1; \end{array}\\ 0, & \mathrm{otherwise}, \end{array}\right.$$

The shared branches are intended to model modality-invariant common context related to suicide risk. From a clinical perspective, the co-occurrence of negative emotion and cognitive distortion represents a more direct high-risk pattern because it reflects simultaneous affective distress and maladaptive cognition. By contrast, positive emotion without cognitive distortion corresponds to a comparatively non-pathological or lower-risk state. Connecting both patterns allows the shared branches to preserve clinically meaningful polarity structure and contrastive context within the conversation, while temporal adjacency models transitions between these states over time.

After constructing the topological structures of the four graphs, a graph attention layer [34] is adopted for each graph branch to adaptively learn the attention weight of each node, capturing the importance of different segment-level features in suicide risk prediction. All four graph branches adopt an identical two-layer stacked graph attention layer architecture, with the hidden layer dimension uniformly fixed at 256. For each graph branch, the enhanced node feature matrix is obtained after the graph attention operation, and then global average pooling is performed on the node feature matrix to obtain the global feature representation of each graph branch, denoted as $G_{s^{p}}\in R^{1\times d_{g}}$, $G_{t^{p}}\in R^{1\times d_{g}}$, $G_{s^{c}}\in R^{1\times d_{g}}$, and $G_{t^{c}}\in R^{1\times d_{g}}$, respectively, where $d_{g}$ is the dimension of the graph global feature.

The global features of the four graph branches are concatenated to form the final fused feature representation $F_{fusion}\in R^{1\times 4d_{g}}$, which integrates the discriminative information of multi-modal, multi-type, and contextual correlated features. The concatenation process is defined as

(22) $$F_{fusion}=\mathrm{concat}\left(G_{s^{p}},G_{t^{p}},G_{s^{c}},G_{t^{c}}\right)$$

where concat(·) denotes the feature concatenation operation. The fused feature $F_{fusion}$ is input into a fully connected layer to complete the suicide risk classification task (binary classification: suicide risk or no suicide risk). The cross-entropy loss function is adopted as the classification loss to optimize the entire model, which is defined as:

(23) $$L_{cls}=-\frac{1}{N}\sum_{i=1}^{N}\left[y_{i}\log\left(p_{i}\right)+\left(1-y_{i}\right)\log\left(1-p_{i}\right)\right]$$

where *N* is the total number of training samples; $y_{i}\in \left\{0,1\right\}$ is the true suicide risk label of the *i*-th sample (1 for suicide risk, 0 for no suicide risk); $p_{i}\in \left[0,1\right]$ is the predicted probability that the *i*-th sample has suicide risk, output by the fully connected layer.

To achieve the end-to-end joint optimization of feature decoupling, information integrity preservation, and suicide risk discriminative ability, we construct a multi-task constrained total loss function by integrating the classification loss from the graph attention module, the decoupling constraint loss, and the feature reconstruction loss from the hierarchical cascaded decoupling module. The total loss function is defined as the weighted linear combination of the three aforementioned loss terms:

(24) $$L_{total}=\lambda_{1}L_{cls}+\lambda_{2}L_{dec}+\lambda_{3}L_{rec}$$

where $\lambda_{1}$, $\lambda_{2}$, and $\lambda_{3}$ are tunable weight hyperparameters that regulate the contribution of the classification loss, decoupling loss and reconstruction loss, to the total optimization objective, respectively. The values of $\lambda_{1}$, $\lambda_{2}$, and $\lambda_{3}$ are determined via grid search.

## 4. Dataset and Experiments

### 4.1. Long-Duration Speech Suicide Risk Prediction Dataset

The dataset used in this study was collected from real-world call recordings of a psychological support hotline at a tertiary Class A hospital in Beijing from January 2015 to December 2017 [35,36]. All participants completed a 12-month systematic clinical follow-up. The binary label system was constructed based on the history of suicidal behavior during the follow-up period as the gold standard, which is fully aligned with the research task: a label of 1 (high suicide risk) was defined as participants having a clinically confirmed history of suicidal behavior by a psychiatrist during the 12-month follow-up period; a label of 0 (low suicide risk) was defined as participants without any history of suicidal behavior during the same period. A total of 1494 valid samples were finally included in the study, including 746 high suicide risk samples and 748 low suicide risk samples. The duration of a single call ranged from 20 to 120 min, which fully conformed to the call duration distribution of real-world psychological support hotlines. To ensure the rigor, robustness, and clinical relevance of model evaluation, this study adopted a subject-independent stratified 5-fold cross-validation scheme for dataset partitioning and model validation. To avoid subject-level or caller-level data leakage, the dataset split was performed at the level of complete speech samples, where each full speech recording was treated as one sample. All segment-level features extracted from the same recording were kept within the same fold and were never split across different folds. In addition, if multiple recordings belonged to the same caller/subject, they were assigned to the same fold. The five-fold cross-validation was stratified by the binary label to preserve class balance across folds.

### 4.2. Data Preprocessing

During the feature extraction phase, an identical preprocessing protocol was adopted for all speech samples. Specifically, the raw speech signals were resampled to 16 kHz mono-channel format, followed by framing with a 20 ms frame length and a 10 ms frame shift, and each frame was windowed using a Hanning window.

For the two-party conversation scenario of psychological support hotlines, this study adopted the pre-trained model pyannote/speaker-diarization-community-1 (https://huggingface.co/pyannote/speaker-diarization-community-1 (accessed on 14 January 2026)) [30,31,32] to complete speaker diarization, separating the speech signals of the counselor and the help-seeker. After diarization, only valid speech segments of the help-seeker were retained for subsequent analysis, completely eliminating the interference of the counselor’s speech on risk prediction. Subsequently, the retained caller’s speech segments are converted into corresponding text content using the SenseVoice (https://github.com/FunAudioLLM/SenseVoice (accessed on 14 January 2026)) tool.

### 4.3. Implementation Details

Both the training and inference of the model are completed in the following environment: the hardware environment consists of two NVIDIA RTX A6000 48 GB graphics cards, one Intel Xeon Gold 6230 processor, and 512 GB of random access memory (RAM). The detailed training configuration is described as follows: the AdamW optimizer is adopted with an initial learning rate of $1\times 10^{-4}$ and a weight decay coefficient of $1\times 10^{-5}$. The batch size is set to 8, and the maximum number of training epochs is set to 50. To accelerate training and reduce GPU memory footprint, automatic mixed precision (AMP) training is enabled, which dynamically switches between FP16 and FP32 computations during the forward/backward passes while maintaining numerical stability. A Dropout layer with a dropout rate of 0.3 is added after the fully connected layer of the model to prevent overfitting. The early stopping strategy is employed: the training is terminated, and the optimal model weights of the corresponding fold are retained when the weighted F1-score on the validation set does not improve for 10 consecutive epochs. The code is publicly available at https://github.com/songchangwei/ACD_SSRNet_Code (accessed on 19 May 2026).

## 5. Results

### 5.1. Comparison with State-of-the-Art Methods

To comprehensively evaluate the performance of the proposed model, this study conducts a systematic comparison between it and the current state-of-the-art (SOTA) as well as latest relevant methods. We select representative studies in the field of speech-based suicide risk prediction in recent years, including the latest models proposed by Ding et al. (2025) [12], Cui et al. (2024) [20], Amiriparian et al. (2024) [11], Chen et al. (2025) [10], and Song et al. (2024) [18], which have the closest task objectives to this study. Furthermore, we also incorporate recent studies focusing on long speech classification tasks, such as the depression detection models proposed by Yu et al. (2025) [26], Zhou et al. (2025). [25] and Sheikh et al. (2025) [24]. Although these models are not specifically designed for suicide risk prediction, their architectures and methods for processing long temporal speech signals can be transferred to this task, providing an important reference for model performance comparison. It is worth noting that the studies by Ding et al. (2025) [12], Amiriparian et al. (2024) [11], Song et al. (2024) [18], Yu et al. (2025) [26], and Sheikh et al. (2025) [24] are all based on the single speech modality. In contrast, Cui et al. (2024) [20], Chen et al. (2025) [10], and Zhou et al. (2025) [25] are built on the dual speech and text modalities.

Experimental results presented in Table 1 show that ACD-SSRNet achieved the best mean performance across all four core metrics while maintaining stable variability across the five subject-independent folds. Specifically, compared with Zhou et al. (2025), the strongest multimodal baseline, ACD-SSRNet, improved the F1-score from 76.20 ± 0.89% to 78.99 ± 1.18% and the Accuracy from 76.14 ± 0.81% to 78.71 ± 1.20%. Paired two-tailed t-tests across the five folds confirmed that these improvements were statistically significant (F1: t=3.98, p=0.0163; Accuracy: t=4.21, p=0.0136; Precision: t=3.67, p=0.0212; Recall: t=3.89, p=0.0175). These results provide statistical support for the reliability of the observed performance gains beyond point estimates alone.

### 5.2. Ablation Study

#### 5.2.1. Ablation Results of Graph Topological Structure

To validate the rationality and superiority of the proposed pathological prior-guided multi-path graph topological structure, which comprehensively integrates emotion labels, cognitive distortion labels, and temporal adjacency, an ablation study on graph topology construction is conducted. The baseline is the complete ACD-SSRNet with the proposed pathological prior-guided graph topology; four model variants are designed to explore different graph construction strategies: the first variant constructs the graph solely based on temporal adjacency between speech segments; the second variant builds the graph using only feature similarity between segment-level features; the third variant establishes graph edges only according to emotion label consistency; the fourth variant connects nodes merely based on cognitive distortion label consistency.

As shown in Table 2, the full model with the proposed pathological prior-guided graph topology achieves the optimal performance across all metrics. Variants relying on a single correlation criterion (temporal adjacency, feature similarity, emotion label consistency, or cognitive distortion label consistency) all exhibit obvious performance degradation, which confirms that the proposed multi-criterion pathological prior-guided graph topology can effectively capture both the contextual correlations of risk-related information and the long-range temporal dependencies in long-form speech and is critical for accurately locating sparse high-risk segments and enhancing the model’s discriminative capability.

#### 5.2.2. Ablation Study on Clinical Prior Features

To verify the essential contribution of the clinically validated affective and cognitive distortion features that serve as core pathological markers of suicide risk, a dedicated ablation study on clinical prior features is performed. Four experimental setups are designed: the full ACD-SSRNet model that integrates both general acoustic-textual features and clinical prior affective–cognitive distortion features is taken as the baseline; the first variant removes affective features while retaining all other features; the second variant discards cognitive distortion features, which are the key pathological prior in this work; the third variant eliminates both affective and cognitive distortion features, leaving only general acoustic and textual semantic features for modeling.

Quantitative results are reported in Table 3. The full model achieves the best performance on all metrics, confirming that the incorporation of clinical prior features effectively boosts the model’s ability to identify subtle suicide risk signals. Removing cognitive distortion features leads to a more significant performance drop than removing affective features, demonstrating that cognitive distortion features act as the dominant clinical prior for distinguishing high-risk samples. When both clinical prior features are removed, the model suffers a substantial decline in all indicators, which validates that the clinically anchored multi-view feature system constructed in this work is critical for improving the discriminative power and clinical alignment of the suicide risk prediction model.

#### 5.2.3. Ablation Study on Core Module Integrity

To validate the necessity, irreplaceability, and synergistic efficacy of the two core innovative modules (i.e., the Hierarchical Cascaded Decoupling Module and the Multi-branch Graph Attention Module) in the proposed ACD-SSRNet, a systematic ablation experiment on core module integrity is conducted. Four experimental groups are designed: the baseline is the full ACD-SSRNet model integrating both core modules; Variant 1 removes the Hierarchical Cascaded Decoupling Module (HCDM) and directly concatenates the four raw feature modalities for subsequent modeling; Variant 2 discards the Multi-branch Graph Attention Module (MBGAM) and adopts a vanilla LSTM for temporal feature fusion instead; Variant 3 eliminates both core modules, retaining only the basic feature extraction layer and fully connected classification layer.

The quantitative results are presented in Table 4. The full model achieves the optimal performance across all metrics, demonstrating the superior capability of the synergistic core modules in mining discriminative suicide risk signals from long-form hotline speech. Removing either core module leads to an obvious performance degradation, verifying that the Hierarchical Cascaded Decoupling Module effectively eliminates heterogeneous feature redundancy and preserves critical high-risk information, while the Multi-branch Graph Attention Module accurately locates sparse high-risk segments and captures long-range temporal dependencies. When both core modules are removed, the model suffers a drastic drop in all indicators, confirming that the proposed core architectural designs are indispensable for enhancing the clinical discriminative power and prediction reliability of the suicide risk assessment model.

#### 5.2.4. Ablation Study on Internal Substructures of HCDM

While Table 4 validates the necessity of the Hierarchical Cascaded Decoupling Module (HCDM) at the module level, it does not isolate the contribution of its internal substructures. We therefore further conduct a fine-grained ablation study on the internal design of HCDM in the following subsection.

To further verify the individual contribution of each internal substructure in HCDM, we conduct a fine-grained ablation study by removing one component at a time while keeping all other settings unchanged. All ablation variants use the same data split, training strategy, and hyperparameter settings as the full model to ensure fair comparison. Specifically, we examine the effect of removing the first-layer orthogonal decoupling loss $L_{ort}$, the knowledge injection mechanism, the shared-feature consistency loss $L_{\mathrm{consist}}$, the cross-modal private-feature orthogonal loss $L_{priv-ortho}$, the intra-modal shared-private orthogonal loss $L_{intra-ortho}$, and the reconstruction loss $L_{\mathrm{rec}}$, respectively. In addition, to further assess the interaction among the second-layer decoupling objectives, we also report a variant without the entire second-layer decoupling block, where $L_{\mathrm{consist}}$, $L_{priv-ortho}$, $L_{intra-ortho}$, and $L_{\mathrm{rec}}$ are removed together.

The results of the fine-grained ablation study are reported in Table 5. The full ACD-SSRNet consistently achieves the best overall performance, indicating that the effectiveness of HCDM does not rely on a single constraint term or auxiliary mechanism, but on the coordinated action of its internal substructures. Removing the first-layer orthogonal decoupling loss degrades performance, confirming the importance of separating domain-specific clinical priors from general modality features at the early stage. Removing the knowledge injection mechanism also leads to a clear decline, showing that the bidirectional fusion between prior-guided and general representations is beneficial for downstream risk discrimination.

For the second-layer decoupling module, removing any individual constraint term, including $L_{\mathrm{consist}}$, $L_{priv-ortho}$, $L_{intra-ortho}$, or $L_{\mathrm{rec}}$, results in performance deterioration, suggesting that shared alignment, private-feature separation, intra-modal disentanglement, and reconstruction regularization each make non-negligible contributions. Notably, the variant without the entire second-layer decoupling block performs worse than the variants removing only a single second-layer term, indicating that these objectives are complementary rather than redundant. Combined with the “w/o HCDM” results in Table 4, these findings further demonstrate that the two hierarchical decoupling stages interact synergistically and jointly account for the effectiveness of HCDM.

### 5.3. Sensitivity Analysis of Key Hyperparameters

To examine the robustness of the loss design, we conducted a local grid search on the auxiliary loss weights while fixing the classification loss weight to $\lambda_{1}=1.0$. Specifically, $\lambda_{2}$ and $\lambda_{3}$ were varied in {0.1,0.2,0.3}, while all other settings were kept unchanged. We focused on this low-weight region because the decoupling and reconstruction terms serve as auxiliary regularizers and are intended to improve representation learning without overwhelming the primary classification objective.

As shown in Table 6, the best performance is achieved at $\left(\lambda_{1},\lambda_{2},\lambda_{3}\right)=\left(1.0,0.1,0.1\right)$, yielding an F1-score of 78.99% and an accuracy of 78.71%. When either $\lambda_{2}$ or $\lambda_{3}$ increases from 0.1 to 0.2 or 0.3, both F1 and accuracy decline gradually. This trend suggests that overly strong decoupling or reconstruction regularization may over-constrain the feature space and weaken the main classification objective. Overall, the results indicate that relatively small auxiliary weights are sufficient, and the setting (1.0,0.1,0.1) provides the best balance between discrimination and regularization.

For the structural hyperparameters, Table 7 shows that a graph hidden dimension of 256 provides the best balance between representation capacity and computational overhead. Increasing the maximum sequence length from 128 to 256 improves performance by preserving more long-range hotline context, whereas further increasing it to 384 yields only marginal gain and no clear advantage over the default setting. The dropout rate of 0.3 produces the most stable overall performance, suggesting an effective balance between regularization strength and information retention. These results indicate that the adopted default hyperparameter configuration is not arbitrary but is supported by empirical sensitivity analysis.

### 5.4. Parameter Efficiency Analysis

To provide a clearer view of the computational characteristics of the proposed framework, we first analyze the four upstream feature branches in terms of parameter scale and feature-extraction latency. As shown in Table 8, the parameter scale differs substantially across feature models. The speech_general branch based on WavLM contains 315,453,120 parameters, the text_general branch based on RoBERTa contains 355,359,744 parameters, the emotion branch based on Emotion2vec contains 164,048,921 parameters, and the cognitive branch based on CD-RoBERTa contains 325,534,732 parameters.

In terms of feature-extraction latency, the speech_general and emotion branches are the main sources of upstream computational cost, requiring 2225.355 ms and 1379.147 ms, respectively, whereas the text_general and cognitive branches require only 166.035 ms and 107.119 ms. This result indicates that the dominant overhead of the upstream pipeline mainly comes from acoustic representation modeling, while the text-related branches are comparatively more efficient.

For the downstream classifier, ACD-SSRNet itself remains relatively lightweight compared with the upstream feature encoders. Specifically, the instantiated ACD-SSRNet contains 3,754,241 trainable parameters. Under a training batch size of 8, the measured training time is 62.436 ms per batch, and the average inference latency is 2.868 ms per sample. These results suggest that the main computational burden of the overall framework lies in multimodal feature extraction rather than in the downstream fusion and classification module. Therefore, the effectiveness of the proposed method is mainly attributed to structured multimodal interaction and graph-based modeling, rather than simply increasing the size of the final classifier.

It should be noted that the reported feature-extraction times only reflect model-side feature computation and do not include audio loading, resampling, segmentation, automatic speech recognition, or caller-speaker selection. Therefore, the above results should be interpreted as an efficiency analysis of the core model components rather than full end-to-end deployment latency.

### 5.5. Interpretability Analysis and Expert Evaluation

To examine whether the model focuses on clinically meaningful evidence, we conducted a qualitative interpretability analysis on five representative cases. For each case, utterance-level transcript fragments were ranked according to the attention scores produced by the model. Specifically, the attention weights associated with each utterance node were aggregated and used as an importance score, and the top-5 fragments with the highest scores were selected as the key evidence.

An expert review was further performed to assess whether the highlighted fragments were consistent with clinically relevant suicide-risk cues, such as explicit suicidal ideation, hopelessness, worthlessness, severe self-blame, and death-related rumination. The expert provided a brief interpretation for each case based on the dominant pattern reflected by the selected evidence.

As shown in Table 9, the highlighted fragments were generally aligned with clinically meaningful risk signals rather than peripheral content. Across cases, the model repeatedly emphasized suicidal intent, chronic despair, self-negation, and hopeless cognition, suggesting that its predictions were supported by interpretable evidence and were broadly consistent with expert judgment.

## 6. Limitations and Future Work

Several limitations of this study should be acknowledged. First, although subject-independent five-fold cross-validation was adopted, the dataset was collected from a single psychological support hotline center in Beijing and contains 1,494 samples. While this cohort provides valuable real-world long-form hotline conversations with follow-up labels, the sample size remains relatively modest compared with the heterogeneity of suicide-related presentations, recording conditions, and interaction patterns that may occur across different populations and service settings. Therefore, the present findings should be interpreted as internal validation on a single-center cohort rather than evidence of full cross-site or cross-population generalizability. Future work should further evaluate the proposed model on multi-center hotlines and clinical cohorts with different populations, recording conditions, and operational workflows.

Second, the present framework was developed and evaluated in a Chinese-language setting. The transcript-based branch relies on Chinese automatic speech recognition output, and the cognitive distortion extractor was built upon CD-RoBERTa fine-tuned on the Chinese SocialCD-3K dataset. In addition, the linguistic expression patterns, cultural context, and help-seeking behaviors reflected in the hotline conversations are specific to the Chinese setting. Therefore, direct transferability of the current framework to other languages, dialects, cultural environments, or populations should not be assumed without additional adaptation and validation.

Third, the text-related components of the framework are potentially affected by upstream preprocessing quality. Speaker diarization errors, overlapping speech, accent variation, background noise, and automatic speech recognition errors may propagate into the transcript-based semantic features and cognitive distortion labels, especially in long, emotionally intense, and acoustically challenging hotline conversations. Although the multimodal design may partially reduce reliance on any single text source, the robustness of the model under transcription uncertainty still requires further investigation.

Finally, clinical safety requires particular caution. The proposed model is intended as a decision-support tool to assist hotline counselors and clinicians, rather than as an autonomous diagnostic or triage system. False negatives may lead to missed high-risk cases, whereas false positives may increase unnecessary alerts and intervention burden. Before real-world deployment, prospective evaluation, threshold calibration, human-in-the-loop review, workflow integration, and continuous safety monitoring are needed to ensure responsible and clinically safe use.

## 7. Conclusions

Speech-based suicide risk prediction is of great clinical significance for real-time screening in psychological support hotlines. However, current methods are limited by insufficient clinical customized features, severe heterogeneous feature redundancy, and misaligned graph modeling with clinical assessment principles, which hinder the detection of sparse high-risk signals in long-form speech. This work presents the Affective & Cognitive Distortions-assisted Speech Suicide Risk Prediction Network (ACD-SSRNet). The model integrates a clinically anchored multi-view feature system with affective and cognitive distortion priors, uses a hierarchical cascade decoupling module to reduce feature redundancy, and adopts a pathological prior-guided multi-path graph attention framework to locate high-risk segments and capture long-range temporal dependencies. Experiments on a real-world hotline dataset show that ACD-SSRNet surpasses state-of-the-art approaches, with relative improvements of 2.79% in F1-score and 2.57% in accuracy. In addition, an attention-based interpretability analysis on five representative cases, together with an expert evaluation, provides preliminary qualitative evidence that the model attends to clinically plausible affective and cognitive cues. Several limitations should be noted. The evaluation was conducted on a single-center Chinese hotline cohort, and external validation on an independent hotline or clinical dataset was not feasible within the current study because access to an additional comparable cohort would require separate inter-institutional ethics approvals, data-access authorization, and harmonized longitudinal follow-up protocols for highly sensitive hotline recordings. Future work will pursue multi-center collaboration, external validation under appropriate ethics approvals, larger-scale blinded expert evaluation, and prospective deployment studies in real-world hotline settings.

## Figures and Tables

**Table 1:** Comparison with state-of-the-art methods. Results are reported as mean ± standard deviation across five subject-independent folds.

Modal	Methods (Year)	F1 (%)	Accuracy (%)	Precision (%)	Recall (%)
A	Ding et al. (2025) [12]	67.62±1.12	68.19±1.01	66.31±1.03	68.98±0.97
A	Amiriparian et al. (2024) [11]	67.07±0.82	67.15±0.83	64.70±0.79	69.62±0.85
A	Song et al. (2024) [18]	69.05±0.93	70.53±0.81	61.11±1.02	79.36±0.92
A	Yu et al. (2025) [26]	70.81±0.89	69.35±0.90	66.20±1.05	76.12±1.01
A	Sheikh et al. (2025) [20]	67.47±0.71	68.37±0.69	67.13±0.91	67.81±1.02
A+T	Cui et al. (2024) [20]	73.68±0.96	75.04±0.93	74.87±0.98	72.52±1.04
A+T	Chen et al. (2025) [10]	74.64±0.95	74.51±0.93	71.25±0.95	78.39±0.98
A+T	Zhou et al. (2025) [25]	76.20±0.89	76.14±0.81	73.31±0.84	79.33±0.82
A+T	ACD-SSRNet	**78.99 ± 1.18**	**78.71 ± 1.20**	**76.39 ± 1.24**	**81.78 ± 1.16**

**Note:** Bold values indicate the best performance among all compared methods.

**Table 2:** Ablation results of graph topological structure.

Model Variants	F1 (%)	Accuracy (%)	Precision (%)	Recall (%)
Full ACD-SSRNet	**78.99**	**78.71**	**76.39**	**81.78**
Temporal Adjacency Only	75.17	76.77	70.32	80.74
Feature Similarity Only	76.03	76.48	71.69	80.93
Emotion Label Consistency Only	77.39	77.62	73.94	81.19
Cognitive Distortion Label Consistency Only	77.20	76.85	74.21	80.45

**Note:** Bold values indicate best performance. Underlined values indicate second-best.

**Table 3:** Ablation Results of Clinical Prior Features.

Model Variants	F1 (%)	Accuracy (%)	Precision (%)	Recall (%)
Full ACD-SSRNet	**78.99**	**78.71**	**76.39**	**81.78**
w/o Affective Features	75.06	75.82	71.16	79.42
w/o Cognitive Distortion Features	75.93	76.35	72.53	79.67
w/o Both Clinical Priors	73.93	74.74	70.91	77.23

**Note:** Bold values indicate best performance.

**Table 4:** Ablation results of core module integrity.

Model Variants	F1 (%)	Accuracy (%)	Precision (%)	Recall (%)
Full ACD-SSRNet	**78.99**	**78.71**	**76.39**	**81.78**
w/o HCDM	76.43	76.83	73.54	79.57
w/o MBGAM	75.30	75.91	70.62	80.65
w/o Both Modules	74.52	74.87	70.19	79.43

**Note:** Bold values indicate best performance.

**Table 5:** Fine-grained ablation results on internal substructures of HCDM.

Model Variants	F1 (%)	Accuracy (%)
Full ACD-SSRNet	**78.99**	**78.71**
w/o $L_{ort}$	77.95	77.79
w/o Knowledge Injection	77.58	77.36
w/o $L_{\mathrm{consist}}$	78.23	78.07
w/o $L_{priv-ortho}$	78.05	77.91
w/o $L_{intra-ortho}$	77.86	77.70
w/o $L_{\mathrm{rec}}$	78.37	78.19
w/o second-layer decoupling	77.18	77.02

**Note:** Bold values indicate best performance.

**Table 6:** Sensitivity analysis of the loss weights with $\lambda_{1}=1.0$ fixed. Each cell reports F1/Accuracy (%).

λ	0.1	0.2	0.3
0.1	**78.99/78.71**	78.76/78.50	78.43/78.18
0.2	78.68/78.41	78.52/78.27	78.21/77.96
0.3	78.34/78.09	78.12/77.88	77.86/77.61

**Note:** Bold values indicate best performance.

**Table 7:** Sensitivity analysis of structural hyperparameters.

Parameter Setting	F1 (%)	Accuracy (%)
Graph hidden dimension = 128	78.17	77.95
Graph hidden dimension = 256	**78.99**	**78.71**
Graph hidden dimension = 512	78.74	78.50
Maximum sequence length = 128	77.92	77.69
Maximum sequence length = 256	**78.99**	**78.71**
Maximum sequence length = 384	78.96	78.70
Dropout rate = 0.1	78.48	78.23
Dropout rate = 0.3	**78.99**	**78.71**
Dropout rate = 0.5	78.25	78.01

**Note:** Bold values indicate best performance.

**Table 8:** Parameter scale and extraction latency of the four feature branches.

Feature Branch	Backbone Model	Parameters	Extraction Time (ms)
speech_general	WavLM	315,453,120	2225.355
text_general	RoBERTa	355,359,744	166.035
emotion	Emotion2vec	164,048,921	1379.147
cognitive	CD-RoBERTa	325,534,732	107.119

**Table 9:** Qualitative interpretability results on five representative cases.

Case	Main Pattern	Top-5 Highlighted Evidence	Expert Judgment
C1	Suicidal ideation + global self-blame	“I really want to die”; “I do not want to live anymore”; “every turning point in my life was a mistake”; “everything has been wrong”; “if I die like this …”	High risk; explicit suicidal intent with pervasive self-blame.
C2	Hopelessness + worthlessness	“I am incurable”; “I have no reason to keep living”; “what reason do I have to remain in this world”; “I am useless”; “I cannot find myself”	High risk; strong hopelessness and self-collapse.
C3	Repeated suicidality + chronic despair	“I already have suicidal tendency”; “my suicidal tendency is at 100 points”; “you can only die”; “I will never get better”; “I keep crying”	Acute high risk; repeated suicide-related expressions strongly support the judgment.
C4	Role failure + severe self-negation	“I am not qualified to be a mother”; “I am worthless”; “I really feel unable to go on living”; “I deny myself in everything”; “the family burden is still there”	High risk; persistent self-denial and burden perception.
C5	Retrospective suicidality + death rumination	“when I was about to kill myself”; “I do not know whether I could really die”; “when I want to die, I feel very small”; “I previously had severe suicidal tendency”; “I was pulled back from the edge between life and death”	High risk but context-sensitive; past crisis and current intent should be carefully distinguished.

## Data Availability

The data presented in this study are not publicly available due to ethical, legal, and privacy restrictions. The dataset consists of sensitive psychological hotline recordings, transcripts, and follow-up clinical outcome information. Public sharing of these data could compromise participant privacy and is not permitted under the current ethics approval and institutional data-use agreement. Access to any de-identified derived data may be considered only after approval by the relevant institutional ethics committee and data-governance authority.
